# Gender-Based Violence in the Asia-Pacific Region during COVID-19: A Hidden Pandemic behind Closed Doors

**DOI:** 10.3390/ijerph19042239

**Published:** 2022-02-16

**Authors:** Michiko Nagashima-Hayashi, Anna Durrance-Bagale, Manar Marzouk, Mengieng Ung, Sze Tung Lam, Pearlyn Neo, Natasha Howard

**Affiliations:** 1 Saw Swee Hock School of Public Health, National University of Singapore, National University Health System, Singapore 117549, Singapore; michiko.hayashi@nus.edu.sg (M.N.-H.); manar.marzouk@visitor.nus.edu.sg (M.M.); mung@nus.edu.sg (M.U.); st.lam@u.nus.edu (S.T.L.); pearlyn.neo@nus.edu.sg (P.N.); natasha.howard@nus.edu.sg (N.H.); 2 Department of Global Health & Development, London School of Hygiene & Tropical Medicine, 15-17 Tavistock Place, London WC1E 7HT, UK

**Keywords:** violence against women, domestic violence, gender-based violence, intimate partner violence, sexual violence, sexual and reproductive health, COVID-19, Asia-Pacific

## Abstract

Since the early stages of the COVID-19 pandemic, there have been reports of increased violence against women globally. We aimed to explore factors associated with reported increases in gender-based violence (GBV) during the pandemic in the Asia-Pacific region. We conducted 47 semi-structured interviews with experts working in sexual and reproductive health in 12 countries in the region. We analysed data thematically, using the socio-ecological framework of violence. Risks associated with increased GBV included economic strain, alcohol use and school closures, together with reduced access to health and social services. We highlight the need to address heightened risk factors, the importance of proactively identifying instances of GBV and protecting women and girls through establishing open and innovative communication channels, along with addressing underlying issues of gender inequality and social norms. Violence is exacerbated during public health crises, such as the COVID-19 pandemic. Identifying and supporting women at risk, as well as preventing domestic violence during lockdowns and movement restrictions is an emerging challenge. Our findings can help inform the adoption of improved surveillance and research, as well as innovative interventions to prevent violence and detect and protect victims.

## 1. Introduction

Gender-based violence (GBV), particularly intimate partner violence and sexual violence against women, is a significant public health issue, a severe violation of women’s human rights [1,2,3,4] and a barrier to global socioeconomic development [5]. Even before the COVID-19 pandemic, the World Health Organisation (WHO) estimated that 30% of all women and girls aged 15–49 who had ever been in a relationship had experienced physical or sexual violence by an intimate partner [1], with GBV named as a ‘global pandemic’ [6]. The health impacts of violence on women and their children are significant, including life-altering injuries, disabilities, mental disorders, sexual and reproductive health (SRH) concerns, including sexually transmitted infections and HIV, unplanned pregnancies, and adverse pregnancy outcomes [6]. Decreased access to SRH facilities may prevent women coming forward to receive family planning, which leads to unwanted pregnancies and thus in some cases may lead to unsafe abortion and maternal death, particularly in resource-poor communities. Data suggest that 7.9% of maternal deaths worldwide are due to abortion [7]. Women killed by intimate partners or family members account for 58% of all female homicide victims, and in many regions, including Asia, the home is the most likely place for a woman to be killed [8]. Beyond the impact on women’s lives, the global economic cost of GBV is estimated at US$1.5 trillion, approximately 2% of gross domestic product globally [9].

During humanitarian emergencies, risks of violence, exploitation, and abuse increase, particularly for women and girls [10], while legal protections and social support networks are weakened [11], making it harder to identify and protect victims of violence. The COVID-19 pandemic has impacted the lives of millions, jeopardising financial stability, family relationships, and physical and mental health. In this context, reports of violence against women increased globally since the early stages of the COVID-19 pandemic, with concerns raised by media, government, non-governmental organisations (NGOs), and advocates around the world [12,13,14,15,16]. Calls to helplines increased by more than 25% in countries such as France, Argentina, Cyprus, and Singapore during lockdown periods [17], and in the UK, calls to the domestic violence hotline increased by 25% within the first week after implementation of strict social distancing measures [18]. In India, complaints related to violence against women after the nationwide lockdown imposed in March 2020 increased by 100% by early April [19]. A recent US study found that increasing average time spent at home significantly increased reported domestic violence [20]. Measures to mitigate the spread of COVID-19, such as quarantine orders, lockdowns, work-from-home arrangements, and school closures clearly threatened many women and girls for whom home was not a safe space [14,21,22,23].

Despite its grave societal consequences and concerns raised around the world, GBV, and especially intimate partner violence (IPV) in the context of infectious disease outbreaks, is still under-researched in many countries due to operational limitations and socio-cultural norms. In the Asia-Pacific region, some region-specific reports have been published [24] and a few country-specific statistics were provided from South Asia [19,25]. However, a huge gap still remains and published empirical studies capturing the situation of GBV in the context of this region are scarce. In view of the above, we aimed to understand the extent of the issue in the region and effects of COVID-19 on GBV, through the perspectives and the phenomena as observed by the SRH and GBV experts in the Asia-Pacific region. As the situation continues to evolve and new pandemics threaten, our findings can inform policymakers’ efforts to address this ‘shadow pandemic’ [17] as part of mainstream pandemic responses in the region as well as globally. 

## 2. Materials and Methods

### 2.1. Study Design

We used a qualitative study design, with a feminist perspective, drawing from semi-structured interviews with SRH experts working in the Asia-Pacific region. Our research question was “What are the reported effects of the COVID-19 pandemic on GBV, including risks and support services for women and young people?”

### 2.2. Sampling and Recruitment

We employed purposive sampling, which is a non-random sampling technique that does not require a set number of participants who are able to provide the required information by virtue of knowledge or experience [26]. Within a range of various purposive sampling methods, we chose expert sampling, which is commonly used when observational evidence is lacking [27]. Participants were recruited to provide regional and country views from service delivery to policymaking (Table 1). The regional samples included SRH experts purposively recruited from UN agencies, international and national NGOs, ministries of health, and academia across twelve countries (i.e., Bangladesh, Cambodia, Indonesia, Laos, Malaysia, Mongolia, Myanmar, Nepal, Pakistan, Philippines, Timor-Leste, Vietnam), selected based on perceived SRH need as determined by UNFPA Asia-Pacific Regional Office. Interviewee inclusion criteria were: (i) resident in a study country and working in SRH; (ii) aged over 21 and able/willing to provide written informed consent; and (ii) working English fluency. The national samples included SRH service providers purposively sampled and recruited in Cambodia (i.e., Phnom Penh and two rural provinces, Mondulkiri and Kampong Cham) in provincial health departments, operational districts, referral hospitals, health centres, and village health committees. Eligibility criteria included: (i) having provided SRH services for at least 6 months before interview; (ii) able/willing to provide written informed consent; and (iii) working Khmer fluency. 

### 2.3. Data Collection

Our interview guides focused on how components of SRH service needs and provision were affected by COVID-19. GBV was mentioned in interviews as it is addressed as part of SRH programmes and further questions were asked on the topic. In the first phase, the researchers of KHANA, an NGO working on HIV/AIDS in Cambodia, conducted 21 phone interviews with SRH providers in Phnom Penh city, Mondulkiri, and Kampong Cham provinces in July–August 2020. Interviews lasted between 40 and 80 min, were audio-recorded and transcribed in Khmer, then translated into English for analysis. In the second phase, which was conducted across the Asia-Pacific region, LST, MH, PN, and NH conducted 26 online interviews in English via Zoom or WhatsApp platforms between November 2020 and January 2021. Interviews lasted 30 to 80 min, were audio-recorded and transcribed professionally. Names were not recorded, and each participant was given the option not to be quoted.

### 2.4. Analysis

We analysed data thematically, using deductive and inductive coding [28]. ADB and MM initially coded the 28 regional transcripts. As GBV was a major emergent theme in the initial analysis, MH further analysed regional data inductively for GBV using NVivo 12 data analysis software (QSR International, 2018, Melbourne, Australia), identifying subthemes, and categorised. MH then coded the 21 transcripts from Cambodia using the same set of GBV-related codes and sub-codes identified in regional transcripts. All codes were reviewed constantly and broad themes and sub-themes were developed through categorisation of these codes in hierarchical structures.

We further used the socio-ecological framework of violence, adopted by major organisations working on this area to promote a multi-faceted approach [29,30,31,32] to guide our analysis, as we organised the themes and interpretation of our findings by (i) individual; (ii) interpersonal/relationship; (iii) community; and (iv) societal levels. The socio-ecological framework has been adopted by WHO and other organisations [29,30,31,32] to understand the aetiology of abuse of children and violence against women. In this figure, we used themes identified in this study to add factors and phenomena specific to the COVID-19 pandemic, such as ‘lockdown/confinement with partner’, ‘reduced community network and activities’ and ‘school closures’.

Using the framework helped to understand the inter-related multi-level factors that put people at risk of becoming victims of violence, and the enabling factors and environments allowing abusers to perpetrate violence [33]. 

## 3. Results

We identified three overarching themes: (i) increasing GBV incidence; (ii) underlying factors for GBV during the pandemic; and (iii) reasons for reduced GBV/SRH service access, use, and quality. We categorised 14 sub-themes under these three themes, two of which were coded deductively (i.e., ‘increased reports of GBV’ and ‘economic strain’) while the rest were coded inductively. We reported the first theme separately, while themes (ii) and (iii) were incorporated into and reported under the socioecological framework.

### 3.1. Increasing GBV Incidence

#### 3.1.1. High Prevalence of GBV before the Pandemic

Most participants noted that GBV was a major issue for women and girls, particularly in countries such as Mongolia, Myanmar, and Pakistan before the pandemic.

“One out of three women in Mongolia experiences some kind of violence each year. So it’s very high, and … one out of two women have experienced any kind of violence in a lifetime.”

(MN1F)

“So in a male-dominated society where 60% of women were already suffering from one or the other type of GBV. This is going to be much higher now.”

(PK1F)

#### 3.1.2. Increased Reports of GBV: The ‘Shadow Pandemic’

Participants across countries explicitly described the overall increase in numbers of calls to helplines and of police reports of physical violence against women since the pandemic began, although empirical data are still to be collected.

“Globally it has increased, even in Malaysia. I think there were more than 2000 cases of domestic violence reported by the women’s organization.”

(MY1F)

“We know that GBV in terms of the incidence has increased significantly. And especially in the case of Mongolia, we know that compared to the same period of last year, this year by end of October incidents of GBV increased by 44%.”

(MN2F)

#### 3.1.3. GBV Affects Society: Absenteeism Due to Violence

The economic impact of GBV was directly felt in sectors where women are a critical part of the workforce. An interviewee in Mongolia referred to a private company trying to address the issues of domestic violence as it was leading to high turnover and absenteeism among female employees, negatively affecting their business.

“I talked to this CEO of this company; they are very interested in this because they are impacted by domestic violence. There’s a very high turnover of female employees because of domestic violence. They don’t sometimes show up, really impacting their business. So they want to address this concern.”

(MN1F)

### 3.2. Underlying Factors for Increased GBV and Reduced Access to Quality Services

**Figure 1:**
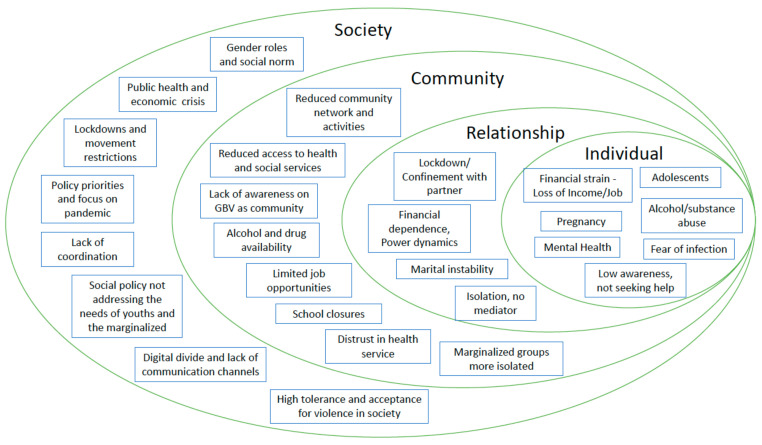
Adapted socio-ecological framework for the GBV during COVID-19 pandemic in the Asia-Pacific region.

We identified underlying risk factors associated with the pandemic and public health measures, and categorised them by four different levels in the adapted socioeconomic model shown in Figure 1 [21,22,23,24], which includes pandemic-specific factors in addition to pre-existing risk factors for GBV.

#### 3.2.1. First Level: Individual

##### Anxiety from Economic Strain

The COVID-19 pandemic affected many people’s livelihoods and family income. Increased psychological stress from loss or reduction of income among men was cited by several participants as an underlying cause of emotional distress that often led to violence.

“Because of the anxiety and the tension and unemployment and where to go and from where to bring the money to home and all this, the men just express their anger on the ladies at home.”

(PK1F)

##### Increased Drug and Alcohol Use

Both male and female interviewees from multiple countries highlighted excessive alcohol consumption and use of drugs among male partners during economic and other stress during COVID-19 pandemic as significant underlying factors triggering violence against women and domestic violence.

“And there’s so much drug abuse, more than alcohol. There’s a lot of illicit drugs that are being used in Pakistan. And with the people losing jobs and depression, all that has gone up.”

(PK2M)

“Even though the government banned the sale of alcohol, and alcoholism is also a big issue in Mongolia, men drink a lot […] and they lose control by drinking alcohol.”

(MN1F)

##### Adolescents and Children

Participants mentioned that young women and girls are more vulnerable to violence within home settings, and their vulnerability increased during the pandemic due to school closures and movement restrictions.

“When I go to a one-stop service centre, I see more girls than the grown-ups. They are basically the victims of sexual violence by a family member, usually like step-father or uncle or grandfather.”

(MN1F)

Economic strain on families due to the pandemic has led to an increased risk of parents opting to marry off their children or adolescents possibly resorting to transactional sex to earn money.

“But the problem was through the economic impact where you see the girls dropping out and that spiking up girls and pregnancy and marriage rates.”

(LA1F)

##### Pregnant Women at Higher Risk

In recent years, there has been increased attention on intimate-partner violence (IPV) during pregnancy, based on its prevalence, adverse health consequences and intervention potential. One participant referred to an increase in ‘incidental maternal deaths’ due to IPV against pregnant women, and also suicide cases among pregnant women.

“Actually, the centre for forensic medicine reported us five suicide cases of pregnant women, which they said that’s nearly unprecedented […] And we suspect that there had been another three maternal deaths this year due to violence… intimate partner violence… these cases, and so we see the increased number of incidental maternal deaths.”

(MN3M)

##### Discrimination and Stigma of Marginalised Groups

Participants in some countries noted that various vulnerable sub-populations of marginalised women, such as ethnic minorities, disabled, poor, sexual minorities, and those living with HIV, whose access to quality SRH services was limited even in the pre-pandemic setting. Participants anticipated that the situation for these women had likely worsened during the pandemic.

“We have anecdotal evidence from different countries, that internally-displaced people, marginalised people, young people, unmarried people, people with different orientations and so on, […] maybe even sex-workers and others, they all have some level of stigma, and it varies, but they are the ones who need to be reached out to because the level of discrimination is pretty high.”

(MY1F)

#### 3.2.2. Second Level: Relationships

##### Isolation and Confinement with Perpetrators

When the COVID-19 pandemic started, countries implemented public health measures to contain its spread, including lockdowns, quarantines, work-from-home arrangements, school closures and restrictions on public transport. These measures increased people’s time at home, potentially prolonging contact with aggressors. Further compounded with isolation from existing social networks due to physical distancing measures, many women became more susceptible to domestic violence.

“On GBV, as in all countries globally, because of the lockdown and the fact that women were trapped in their families and often with their abusers, there was an increase in domestic violence, a very high increase in domestic violence. At the same time, women had no access to the support system that exists in the country.”

(NP1F)

##### Unable to Seek Help

Interviewees reported that victims of violence do not seek help even if services were available. Reasons for this hesitancy are multi-faceted, some of which, such as constant monitoring and pressures imposed by the perpetrating partner, were reinforced during the pandemic.

“Most of them tried to hide it actually. When they come and complain about what makes them in pain, I casually ask them, and they eventually tell me that their husband has physically abused them. […] because most of them won’t tell us. Some of them are brought here by their husband, and they don’t dare to say anything, but we can observe and see that they are having pressure from their husband.”

(KH11UF)

Living under scrutiny by perpetrators during lockdowns and work-from-home hindered women’s access to helplines, counselling, and shelter services.

“And in fact, what the police records show is that in the initial period, the number of calls in the police helplines reduced. And that is clearly because women who are in lockdown do not have the privacy to make those calls in safety. So those calls reduced, and that was not so much a signal that there’s less GBV, but it was because of the context. And then when the lockdown was eased, I think they started receiving more calls.”

(NP1F)

#### 3.2.3. Third Level: Community

##### SRH Services Short-Staffed Due to COVID-19 Response

Health services, especially SRH services that many women visit regularly, and GBV services, such as one-stop crisis centres, play a critical role as the first contact point to identify women at high risk, address their physical and mental needs, and protect them from further harm. Although SRH services remained functional in most study countries during the first year of the pandemic, many participants acknowledged declines in the quality of SRH services, resulting from understaffing to prioritise COVID-19 services.

Issues of limited human resources for health were apparent, primarily due to task shifting, with SRH staff assigned to pandemic-related services, while the remaining health services including GBV services were de-prioritised. The low capacity of such facilities was also noted as an issue prohibiting wider accessibility of services.

“COVID definitely stopped non-essential health services. […] Especially, if it is domestic violence, the people will still think this is their own problem, instead of this is the government or the country problem…”

(MM2M)

Lockdowns complicate women’s access to family planning services. When health facilities are closed, supplies cannot reliably get through, community midwives cannot attend women, and some women are forced to have unsafe abortions with little to no aftercare available. This inevitably leads to more unwanted pregnancies, as highlighted by an interviewee in Pakistan:

‘‘When there is no supplies available, when there is no access to the supply for supplies, when there is a lockdown and then there’s an access issue there is a fear that there will be a lot of unwanted pregnancies.’’

(PK2M)

##### Fear of Infection among Service Providers

Similarly, access to services for GBV survivors—such as one-stop crisis centres that provide shelter, injury care, and psychosocial counselling—were disrupted by the pandemic. In the initial stage, service providers were reportedly afraid to keep doors open to potentially-infected women. GBV survivors also feared they might be infected in such facilities.

“What we also realized is that staff working in those shelter homes—one-stop service centres, they were afraid to have a client, you know? They closed it because it was a new disease for them and then they were afraid to get COVID.”

(MN2F)

##### Lack of Information about Helplines

Some interviewees noted that even if hotlines were set up and made available, information on such services was not disseminated sufficiently to reach all at-risk women.

“In Vietnam now we have the hotline; one hotline that people with violence can call. […] But people have to know the number…”

(VN2M)

##### School Closures Affecting the Safety of Children and Adolescents

In many countries where schools serve as a safe haven for children and youths at risk, school closures due to COVID-19 pandemic led to heightened risk of violence including child abuse targeting both girls and boys.

“During the lockdown what we had is like the boys and girls having to spend more time in households, they were more exposed to this domestic violence.”

(LA3F)

##### Distrust in Healthcare Services

Multiple participants highlighted women’s lack of trust in available healthcare due to poor quality services or disrespectful staff. Coupled with poor accessibility, lack of information, lack of self-esteem and often the added burden of financial barriers, women were discouraged from seeking help at health services.

“And then also people not trusting the health facilities, not trusting the quality of health services you receive…”

(TL1F)

#### 3.2.4. Fourth Level: Societal

##### GBV Not Prioritised during Pandemic Response

In many Asia-Pacific countries, availability of quality services and prevention programmes depends largely on public funding and governmental priorities. Countries with limited resources often placed low priority on GBV prevention pre-pandemic, and GBV policies and programmes remained neglected during the pandemic response.

“You don’t even see a GBV-related department in any of the government systems. […] That might not be like intentional, because the staff are not that many, and especially if the COVID come up right? […] I think they recently launched the GBV guiding manual, maybe two to three years ago. But you know, that uptake of this, the whole GBV as an essential service, is not there yet.”

(MM2M)

##### Culture and Social Norms

Cultural sensitivity and sense of shame associated with being a victim of violence hinder women from reporting or seeking help, especially in various Asian cultures. While their familiar social networks and access to existing services were limited due to pandemic, women found it even more difficult to reach out to helplines or other services that are unfamiliar to them.

“Women here that face sexual abuse, it’s not easy to solve. It’s not easy to tell, they are still shy… If there is such a case, it needs months, then they tell us, they don’t know what to do.”

(KH14RF)

## 4. Discussion

This study is an initial effort to examine the accounts of regional SRH experts, with both health policy and clinical experience, on GBV issues both before and in the first year of the COVID-19 pandemic. These insights are based on deep understanding of specific country contexts, which enabled us to consider determinants of violence and barriers to seeking help, as direct and indirect consequences of the pandemic and mitigation measures imposed. Several risk factors specific to the context, such as rapid economic downturn, lockdown and confinement at home, school closures, isolation from social networks and the disruptions in the provision and utilisation of health and social services were identified, on top of pre-existing risk factors for violence against women in vulnerable situations. New reports and insights on increased GBV globally support our findings [12,34,35]. Many risk factors are interrelated across different levels in the socio-ecological framework. We argue that understanding the dynamics of multiple risk factors using the adapted socio-ecological framework helps us to envisage the influence of future health emergencies on the vulnerabilities of women and girls.

At an individual level, youth, lack of education, and low socio-economic status are known as risk factors for the violence [34,36,37], and the vulnerabilities multiplied for many women and girls as the pandemic unfolded. These include economic strain, which is strongly associated with psychological wellbeing and violence against women in cohabiting relationships [38,39], specifically the employment status of men [40,41]. Loss of income for women in abusive situations increases the difficulty of escape [17,42]. Adolescents face an added layer of risks when their parents lose income. Increased alcohol abuse is known to be strongly associated with violent behaviours [43,44], and a study in the USA showed a significant increase in alcohol consumption since the onset of COVID-19 [45]. There has been a call for a deliberate and collaborative response among government and industry actors to assist individuals to limit alcohol use as a means of coping with pandemic stressors [46].

At relationship and community levels, women and girls’ vulnerability in health crises is further exacerbated by isolation from family and friends [47], financial dependence due to loss of jobs, lack of access to regular social networks and sources of social support, disruptions of health and other support services [48]. COVID-19 mitigation measures created an environment where GBV perpetrators can remain unchecked, leaving their victims to cope in silence. For adolescents and children, school closures meant they were cut off from their usual social network and protection mechanisms, rendering them more at risk and less obvious if they became victims of violence, exploitation, or even child marriages. Child marriage and trafficking were identified as effects of the 2013–2015 Ebola epidemic [49], and it is estimated that up to 10 million more girls will be at risk of becoming child brides over the next decade as a result of COVID-19 [50]. In addition to children and youth, marginalised groups such as ethnic minorities, disabled, the elderly, and LGBTQI were noted in our interviews as especially vulnerable and often neglected in health services and communications outreach. 

On top of decreased social support in communities, routine accessibility and utilisation of SRH and GBV services were significantly reduced due to fear of infection among service-users and health-workers, as well as staff shortages as resources were diverted to pandemic response. The vulnerability of pregnant women may have increased due to reduced access to regular antenatal services, and a lack of safe abortion facilities. As SRH services often provide first-line support and valuable opportunities for detecting hidden GBV cases, increased awareness among the healthcare staff and provision of supportive care in safe settings should be considered essential during pandemic responses in all countries [51,52].

Given the complexities of violence during the pandemic, it is vital to explore alternative means of identifying and protecting victims and to establish communication channels to ensure no woman or girl is left completely isolated, especially for those without any digital means to communicate or discreetly request help. Some successful examples of innovative detection and protection programmes globally provide hope, such as use of certain codes or colours as SOS messages in public spaces such as pharmacies, use of a disguised free phone application to report violence to police or emergency services, or even training and mobilising postal service staff to identify signs of violence when visiting homes for delivery service [21,53]. A UK government initiative to link with private pharmacies to launch a nationwide codeword scheme to identify and help victims of violence is one example of how policymakers could mobilise existing infrastructure in communities to ‘to give some of the most vulnerable people in society a critical lifeline’ [54].

This pandemic demonstrated that inequities are often reinforced in times of crisis. Social norms of gender inequality and acceptance of routine violent behaviours persisted within households during lockdowns, where no outsiders, including friends or families, were able to reach out to at-risk people. Through the interviews, we saw a participant understating violence as “it’s just a fight in the family” (KH13UF), even among the health-workers responsible for detecting cases. In this context, continuous training of frontline workers on GBV across all levels, and strong messaging through public campaigns are critical in influencing social perceptions surrounding gender norms and violence. Such public messaging should be incorporated as an essential part of pandemic planning.

The Inter-Agency Standing Committee notes that humanitarian action in crises is most effective when focused not only on meeting immediate needs of those directly affected, but also on protecting the rights and long-term wellbeing of the most vulnerable people at every stage [10]. It is essential for governments to recognise that GBV in their communities not only violates and traumatises its survivors, but also leaves its victims with a higher prevalence and severity of physical and mental disorders [55,56,57], with longstanding effects on the resilience, health and productivity of societies. Women’s wellbeing impacts their children and other family members under their care, and at the same time women are vital members of workforces in many sectors, such as the garment factories mentioned in our interviews. Policymakers are urged to recognise it as a public health issue for its far-reaching consequences at the population and societal levels, and to address violence and underlying gender inequalities as barriers to sustainable socioeconomic development that require multi-level and multi-sectoral approaches. In addition, future research could examine this topic using a quantitative approach, analysing any association between the factors we have identified and GBV incidence.

## 5. Conclusions

Violence can result from a combination of influences on behaviour and can easily be exacerbated during crises such as the COVID-19 pandemic. Identifying and supporting women at greater risk, as well as preventing violence within homes during lockdowns and movement restrictions is an emerging challenge. A multi-disciplinary public health approach is required, mobilising not only health and judicial sectors, but also education, social service, private sector, and community leaders in preventing and halting violence.

This study presents compelling evidence of widespread violence against women and girls in the Asia-Pacific region during the COVID-19 pandemic. Analysing the emerging themes using the socio-ecological framework allows us to see the complexity and multi-layered approach that is required to address GBV during the pandemic. However, data on the extent of the problem are still scarce. Noting that most people who experience intimate partner violence do not seek help [58], further studies and strengthened surveillance systems are necessary to fully understand the prevalence and the dynamics specific to respective communities. Mainstreaming GBV service provision and preventive measures in socio-economic response to pandemics is crucial [59] and in doing so, much can be learned from practical and innovative approaches taken so far [17,21,60], including success stories and lessons learned from around the world.

## Figures and Tables

**Table 1:** Presents characteristics of the interview participants.

ID	Country	Sector	Professional Role	Gender
BG1M	Bangladesh	UN	Health System Specialist	Male
BG2M	Bangladesh	INGO	Country Director	Male
KH1M	Cambodia	UN	Programme Director	Male
KH2M	Cambodia	NGO	Country Director	Male
ID1F	Indonesia	UN	SRH Programme Specialist	Female
ID2F	Indonesia	UN	Adolescent SRH specialist	Female
LA1F	Laos	UN	Country Representative	Female
LA2F	Laos	UN	SRH Specialist	Female
LA3F	Laos	INGO	Head of Adolescence Programme	Female
MY1F	Malaysia	UN	Assistant Representative	Female
MY2M	Malaysia	INGO	Director SRH programme	Male
MN1F	Mongolia	UN	Head of Office	Female
MN2F	Mongolia	UN	Assistant Representative	Female
MN3M	Mongolia	UN	SRH Programme Specialist	Male
MM1M	Myanmar	UN	SRH Programme Specialist	Male
MM2M	Myanmar	UN	Programme Director	Male
NP1F	Nepal	UN	Representative	Female
NP2F	Nepal	UN	Assistant Representative	Female
NP3F	Nepal	MOH	Consultant	Female
PK1F	Pakistan	MD	Medical Director/Obstetrician	Female
PK2M	Pakistan	UN	Technical Specialist	Male
PH1F	Philippines	UN	Assistant Representative	Female
PH2F	Philippines	INGO	SRH Programme Specialist	Female
PH3F	Philippines	DOH	Programme Director	Female
TL1F	Timor-Leste	INGO	Country Director	Female
TL2M	Timor-Leste	INGO	Programme Director	Male
VN1F	Vietnam	NGO	Founder	Female
VN2M	Vietnam	UN	SRH Programme Specialist	Male
KH03RF	Cambodia	Provincial Health Department	MCH Senior Management	Female
KH04UF	Cambodia	Provincial Health Department	MCH Senior Management	Female
KH05RM	Cambodia	Provincial Health Department	MCH Senior Management	Male
KH06UM	Cambodia	Operational District	MCH Senior Management	Male
KH07RM	Cambodia	Operational District	MCH Senior Management	Male
KH08RF	Cambodia	Referral Hospital	Obstetrics Senior Management	Female
KH09RF	Cambodia	Referral Hospital	Obstetrics Senior Management	Female
KH10UF	Cambodia	Referral Hospital	Midwife	Female
KH11UF	Cambodia	Health Centre	Midwifery Senior Management	Female
KH12UF	Cambodia	Health Centre	Obstetrics	Female
KH13UF	Cambodia	Health Centre	Midwife	Female
KH14RF	Cambodia	Health Centre	STI Consultant	Female
KH15RF	Cambodia	Health Centre	Obstetrics Senior Management	Female
KH16RF	Cambodia	Health Centre	Obstetrics Senior Management	Female
KH17RF	Cambodia	Health Centre	Midwife	Female
KH18RM	Cambodia	Community	Village Health Support Group	Male
KH19RF	Cambodia	Community	Village Health Support Group	Female
KH20UF	Cambodia	Community	Village Health Support Group	Female
KH21UF	Cambodia	Community	Village Health Support Group	Female
KH22RF	Cambodia	Community	Village Health Support Group	Female
KH23UF	Cambodia	Community	Village Health Support Group	Female

## Data Availability

Transcript data supporting the findings of this study are available from the corresponding author upon request.
